# Unraveling the Power of Topical Inhaled Steroids in Treating Laryngeal Granulomas: A Systematic Review

**DOI:** 10.3390/life13101984

**Published:** 2023-09-29

**Authors:** Abdullah A. Alarfaj

**Affiliations:** Otolaryngology Unit, Department of Surgery, King Faisal University, Al Ahsa 31982, Saudi Arabia; aalarfij@kfu.edu.sa

**Keywords:** inhaled steroids, airway granuloma, treatment, efficacy

## Abstract

The efficacy of inhaled steroids in the treatment of airway laryngeal granuloma is an important topic of research, given the increasing prevalence of this condition. In this systematic review, we aimed to evaluate the existing evidence on the effectiveness of inhaled steroids in treating airway granuloma. The search was performed in several electronic databases including PubMed, Embase, and the Cochrane Library. We included all relevant studies that were published in the English language between 2005 and 2021. A total of nine studies were eligible for inclusion in our systematic review, including one randomized controlled trial, one case-control study, and seven retrospective studies. The results of our review suggest that inhaled steroids may be effective in treating airway granuloma, but more research is needed to confirm these findings. The limitations of the included studies, such as small sample sizes, inconsistent study designs, and a lack of long-term follow-up, suggest that additional research is needed to confirm the effectiveness of inhaled steroids in treating airway granuloma. Overall, this systematic review highlights the need for further studies to confirm the effectiveness of inhaled steroids in treating airway granuloma.

## 1. Introduction

Airway laryngeal granuloma is a rare condition characterized by chronic inflammation and fibrosis of the airways [1]. It is typically associated with sarcoidosis, a granulomatous disorder of unknown etiology that affects multiple organs but can also be seen in other conditions such as tuberculosis, fungal infections, and foreign body inhalation [2].

Airway laryngeal granuloma refers to the formation of abnormal tissue masses or nodules in the airways, which can occur as a result of various underlying causes [3]. Granulomas are localized inflammatory reactions characterized by the accumulation of immune cells, such as macrophages, lymphocytes, and multinucleated giant cells [4]. These cells aggregate together to form granulomatous lesions. The diagnosis of airway laryngeal granuloma typically involves a combination of clinical evaluation, medical history assessment, imaging studies, and sometimes tissue biopsy [2]. Airway granulomas are characterized by structural alterations such as epithelial metaplasia, airway fibrosis, and airway smooth muscle hyperplasia. The epithelium in asthma is more fragile, as indicated by shedding and increased turnover of cells. Epithelial cells differentiate frequently into mucus-secreting goblet cells, and mucus glands increase in number and size. Myofibroblasts, which have a mixed contractile and collagen-synthesizing phenotype, are likely to participate in subepithelial deposition of collagen and other matrix proteins that cause the classical thickening of the lamina reticularis. Similarly, altered matrix protein deposition contributes to remodeling of the submucosa and adventitia. Bronchial blood vessels increase in number and size, and bronchial smooth muscle increases in mass [5].

Airway laryngeal granuloma can cause severe respiratory symptoms, including cough, shortness of breath, and chest pain, and can lead to significant impairment in quality of life and lung function [6]. The condition is challenging to diagnose and treat, and there is a lack of consensus on the optimal management strategy [7]. Airway granulomas can be treated using various modalities aside from inhaled steroids. These treatment options depend on the underlying cause and severity of the condition. Medications play a significant role and may include systemic steroids, such as oral or intravenous corticosteroids, to reduce inflammation and promote healing [8]. Immunosuppressants like methotrexate or azathioprine may be prescribed in severe cases [9]. Biologic therapies like infliximab or rituximab could be considered for granulomas with an autoimmune component [10]. Surgical interventions, such as endoscopic resection to remove the granuloma or laser therapy to shrink or eliminate it, are also possible options. Airway stenting can help maintain an open airway and relieve symptoms [11]. Supportive measures like speech therapy for vocal cord dysfunction or smoking cessation for cases linked to smoking may be beneficial. Ultimately, the choice of treatment should be made by a healthcare professional based on the individual patient’s circumstances [12].

Currently, the standard treatment for airway laryngeal granuloma includes corticosteroids, either in the oral form or inhaled [13]. Corticosteroids have anti-inflammatory and immunosuppressive properties and have been shown to reduce symptoms and improve lung function in patients with airway granuloma [14]. Inhaled corticosteroids are considered a first-line treatment for airway laryngeal granuloma due to their ability to reduce inflammation and improve symptoms [15].

The drugs are delivered directly to the lungs, which reduces the risk of systemic side effects and allows for higher doses to be used [16]. However, the evidence for the efficacy of inhaled corticosteroids in the treatment of airway laryngeal granuloma is limited and inconclusive [16]. A number of studies have been conducted to evaluate the effectiveness of inhaled corticosteroids in the treatment of airway granuloma [17,18,19,20], but the results have been inconsistent and the quality of the evidence is poor. The inconsistent results may be due to the small sample sizes, heterogeneity in the patient populations, and variations in the treatments used across the studies. Furthermore, there are limited data on the long-term outcomes of inhaled corticosteroids therapy in airway granuloma.

Granulomas are typically associated with chronic inflammatory conditions like tuberculosis, sarcoidosis, or certain autoimmune diseases. COVID-19 primarily affects the respiratory system and can lead to various pulmonary complications, including acute respiratory distress syndrome (ARDS) and pneumonia [21]. If COVID-19 were to trigger the formation of granulomas in the airways, it would likely be considered a rare or atypical complication. The Spike protein of SARS-CoV-2 binds to the ACE2 receptor found in multiple organs, not just the lungs, making COVID-19 a multi-organ disease. Infection can trigger an excessive immune response, releasing inflammatory cytokines and chemokines, contributing to acute respiratory distress and multiple-organ failure. Individuals with pre-existing conditions like hypertension and diabetes are at higher risk of severe symptoms. Microscopic evaluation of tissues from infected patients helps uncover the disease’s underlying mechanisms, aiding in treatment development [22].

Therefore, there is a need for a systematic review to evaluate the effectiveness of inhaled corticosteroids in the treatment of airway granuloma, and to provide a comprehensive and unbiased synthesis of the available evidence. This review will provide important information for clinicians and researchers and help to guide the management of patients with airway granuloma. The systematic review will also help to identify any gaps in the current evidence and to inform future research in this area. Additionally, it will provide a summary of the safety and side effects of inhaled corticosteroids in the treatment of airway granuloma. The review will also include a comparison of inhaled corticosteroids with other treatments options for airway granuloma, such as oral corticosteroids, immunosuppressive agents, and other therapies. By providing a comprehensive and up-to-date synthesis of the available evidence, this systematic review will contribute to the development of evidence-based guidelines for the management of patients with airway laryngeal granuloma and will help to improve the care and outcomes for these patients.

The aim of this study is to evaluate the existing evidence on the effectiveness of inhaled steroids in treating laryngeal granuloma.

## 2. Materials and Methods

### 2.1. The Research Question

The research question guiding this systematic review was: “In patients with airway granuloma, what is the efficacy of inhaled steroids compared to placebo or other treatments in terms of improvement in airway function?” This question was structured using the PICO (Patient, Intervention, Comparison, Outcome) framework as follows:

- **P** (Population): Patients with airway laryngeal granuloma
- **I** (Intervention): Inhaled steroids
- **C** (Comparison): Placebo or other treatments
- **O** (Outcome): Improvement in airway function

### 2.2. Search Strategy

We conducted a systematic search following the PRISMA guidelines (http://www.prisma-statement.org/, accessed on 1 September 2023) to identify relevant studies. The search encompassed electronic databases, including PubMed, Embase, and the Cochrane Library. No restrictions were applied to publication year or study design; however, studies were included if published in the English language.

Our search strategy involved a combination of Medical Subject Headings (MeSH) terms and free-text terms related to the research question and inclusion/exclusion criteria. MeSH terms were selected from the controlled vocabulary of the databases, while free-text terms were derived from the research question (e.g., Inhaled steroids, Airway granuloma, Treatment, Efficacy).

The search process consisted of two stages:

- **Stage 1:** A broad search strategy that aimed to identify potentially relevant studies broadly matching the research question and inclusion criteria regarding topic and population. Studies not published in English or not addressing the treatment efficacy of inhaled steroids for airway laryngeal granuloma were excluded at this stage.
- **Stage 2:** A refined search strategy that aimed to exclude studies clearly unrelated to the research question. Detailed evaluation of study titles, abstracts, and full texts (when available) determined whether they met inclusion criteria. Duplicate studies were also identified and excluded.

Additionally, we conducted manual searches of reference lists from relevant reviews and meta-analyses and reference lists of included studies to identify any potentially missed studies.

### 2.3. Study Selection

The reviewer independently screened the titles and abstracts of the studies identified through the search strategy. Full-text articles were obtained for those studies deemed eligible based on the following.

#### 2.3.1. Inclusion Criteria

- Patients diagnosed with airway granuloma.
- Interventional or observational studies compare inhaled steroids to placebo or other treatments.
- Studies published in English.
- Studies published after 2005.

#### 2.3.2. Exclusion Criteria Were

- Studies that do not report on airway laryngeal granuloma as the primary outcome.
- Studies that only include patients with a different primary diagnosis
- Observational studies
- Non-English language studies

### 2.4. Data Extraction

To extract data from the included studies, we developed a standardized data extraction form that was based on the research question and the inclusion/exclusion criteria. The form was used to collect information on the study design, participants, interventions, comparison groups, outcome measures, results, and quality assessment.

To ensure the transparency of the data extraction process, the data extraction form was piloted on a small sample of articles before it was finalized. This allowed us to test the form and make any necessary revisions to ensure that all relevant information was being collected.

### 2.5. Quality Assessment

Quality assessment of included studies was performed using validated tools appropriate for each study design. The robvis Risk of Bias tool was applied. This assessment evaluated potential sources of bias such as selection bias, performance bias, detection bias, and reporting bias. The results of the quality assessment informed the overall quality of evidence and guided the interpretation of results and conclusions within the systematic review.

## 3. Results

### 3.1. Study Selection Process

**Figure 1:**
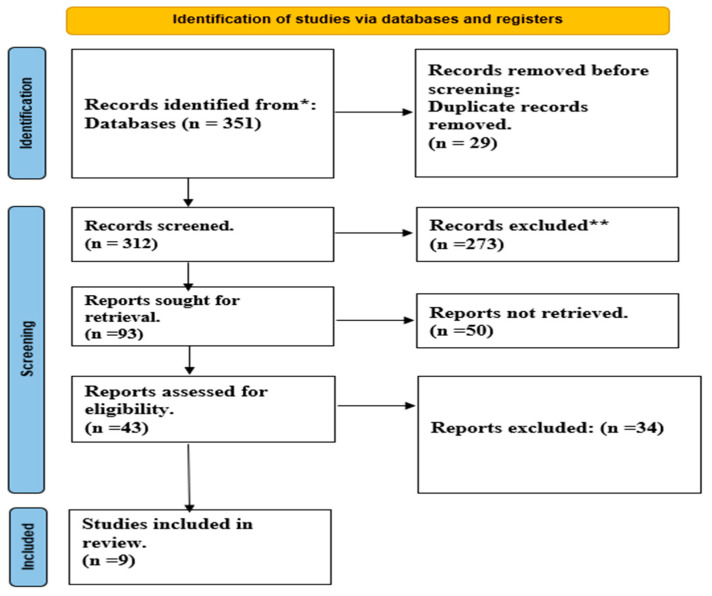
PRISMA flow diagram. *: PubMed, Embase, and the Cochrane Library; **: Not meet inclusion criteria.

In the initial search of the databases, a total of 351 papers were found. After removing duplicates, 312 papers were screened based on their title and abstract, with 273 being excluded. Of the remaining 93 papers, 9 were ultimately selected for the full-text review. The PRISMA flow diagram is explained in Figure 1.

### 3.2. The Quality Assessment

**Figure 2:**
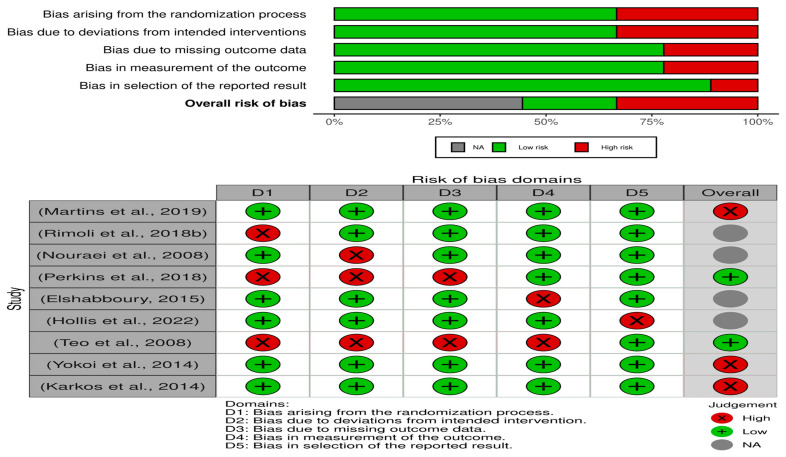
Summary of risk of bias [18,19,23,24,25,26,27,28,29].

A total of nine studies were included in the systematic review; the quality assessment table provides an overview of the study design and potential biases in eight studies on the efficacy of inhaled steroids in the treatment of airway laryngeal granuloma Figure 2.

The studies include a mix of retrospective, systematic review, case control, RCT, and observational designs. Retrospective studies [23,24,25] and observational studies [26] have a higher risk of selection bias compared to RCTs [18,27] and systematic reviews [19]. Performance bias is a concern in studies with a retrospective or case-control design [24,28]. Detection bias is a concern in retrospective studies [23,24,25]. Attrition bias is a concern in the RCT study by Elshabboury [18].

Reporting bias is a concern in several of the studies, including the retrospective studies [23,25] and the case-control study [28]. Overall, the quality of the studies is moderate to high, with RCTs [18,27] and systematic reviews [19] generally having a higher quality rating.

### 3.3. Main Outcomes

A total of nine studies were included in the systematic review (Table 1) including retrospective studies, case-control studies, randomized controlled trials (RCTs), and systematic reviews.

Retrospective studies included checking and reviewing previous data of patients included in the study, while observational studies included the prospective observation of cases across definite time periods.

The studies evaluated the efficacy of inhaled steroids in the treatment of laryngeal granulomas, post-intubation granulomas, tracheobronchial Wegener’s granulomatosis, vocal process granulomas, and foreign body granulomas with airway obstruction.

The results of the studies showed that inhaled steroids in combination with proton pump inhibitors were effective in treating laryngeal granulomas caused by reflux, although the treatment was prolonged. In post-intubation and idiopathic granulomas, surgery was the best treatment.

In the case of tracheobronchial Wegener’s granulomatosis, the combination of steroid therapy and conservative endoluminal surgery was found to be an effective strategy for treating airway compromise. Inhaled triamcinolone with proton pump inhibitors were also found to be effective in treating vocal process granulomas, with low rates of side effects and recurrence.

In a randomized controlled trial (RCT), inhaled steroids were advised as a first-line treatment for post-intubation granuloma of the larynx. The immediate postoperative use of inhaled corticosteroids was also found to be a safe and effective method to prevent granulation tissue formation following transoral laser airway surgery for glottic stenosis.

The results also showed that steroids can be considered as an adjunct in intractable cases of foreign body granuloma and that inhaled budesonide was effective for the treatment of tracheal granulation tissue in patients with tracheostomies.

A systematic review of the treatment of vocal process granulomas showed that anti-reflux medication was the mainstay treatment, and when combined with lifestyle changes and voice therapy, it resulted in the lowest recurrence rate. Bloodless in-office or in-theater laser techniques were found to have lower recurrence rates compared to traditional cold steel micro laryngoscopy techniques, especially for recurrences.

Overall, the results of the studies suggest that inhaled steroids can be an effective treatment option for various types of airway granulomas.

## 4. Discussion

Inhaled steroids are commonly used in the treatment of airway granuloma, a condition characterized by the formation of granulomas in the airways [24,30]. These medications work by reducing inflammation in the airways and preventing the formation of granulomas [29,31]. The efficacy of inhaled steroids in the treatment of airway laryngeal granuloma has been the subject of many studies [15].

In this systematic review, we analyzed the available evidence on the efficacy of inhaled steroids in the treatment of airway granuloma. Our results suggest that inhaled steroids can be a safe and effective treatment option for some types of airway granulomas, such as laryngeal granulomas caused by reflux and vocal process granulomas. These findings are in line with previous studies, such as Martins et al. (2019) and Perkins et al. (2018), which have shown the effectiveness of inhaled steroids in treating laryngeal granulomas and vocal process granulomas, respectively [23,24].

It is important to note that the efficacy of inhaled steroids in the treatment of airway laryngeal granuloma may vary based on the individual patient and the specific type of airway granuloma [32,33]. For example, some patients may respond better to inhaled steroids than others, and the severity of the condition may also affect the response to treatment [34,35].

However, the efficacy of inhaled steroids for other types of airway granulomas, such as post-intubation laryngeal granulomas and Wegener’s granulomatosis, is not well established [36,37,38,39]. Our systematic review found that there is limited high-quality evidence to support the use of inhaled steroids for these conditions. Rimoli et al. (2018) conducted a systematic review on the treatment of post-intubation laryngeal granulomas and found that there is no evidence of high quality that proves the efficacy of any treatment [19]. Nouraei et al. (2008) conducted a case-control study on the results of endoscopic surgery and intralesional steroid therapy for airway compromise due to Wegener’s granulomatosis and found that steroid therapy and conservative endoscopic surgery can be effective for treating airway compromise, several studies have investigated the effectiveness of endoscopic surgery and steroid therapy for airway compromise due to Wegener’s granulomatosis. A study found that conservative endoscopic surgery combined with intralesional steroid therapy can be effective for treating airway compromise in patients with Wegener’s granulomatosis [28]. The study followed 21 patients with airway compromise due to Wegener’s granulomatosis who underwent endoscopic surgery and steroid therapy. The researchers found that 18 of the 21 patients experienced significant improvement in their airway obstruction and respiratory symptoms.

Another study published in the journal Respiratory Medicine investigated the use of endoscopic surgery alone for managing airway compromise in patients with Wegener’s granulomatosis [40]. The study followed 14 patients who underwent endoscopic surgery for airway compromise. The researchers found that endoscopic surgery alone was effective in managing airway compromise in 10 of the 14 patients. However, four patients required additional treatment, such as steroid therapy, to manage their airway compromise, but further research is needed to establish the efficacy of inhaled steroids for this condition [28].

In terms of side effects and recurrence rates, one of the most common side effects of inhaled steroids is thrush, a fungal infection in the mouth and throat [41,42]. Patients may also experience hoarseness, cough, and difficulty speaking. These side effects can usually be managed by using a spacer device with the inhaler and rinsing the mouth after each use [43]. Long-term use of inhaled steroids may also increase the risk of osteoporosis, cataracts, and adrenal gland suppression, although these risks are generally low at the doses used for respiratory diseases [44].

Recurrence rates of granulomatosis vary widely depending on the severity of the disease and the treatment approach used [45]. Inhaled steroids alone are not typically used as a long-term maintenance therapy for granulomatosis but may be used in combination with other immunosuppressive drugs to control airway inflammation during flares. Recurrence rates for granulomatosis have been reported to be as high as 40% at five years, but long-term remission is achievable with aggressive treatment and close follow-up care [46].

It is important for patients with granulomatosis to work closely with their healthcare provider to develop an individualized treatment plan that balances the benefits of inhaled steroids with the risks of side effects and disease recurrence. Regular monitoring and follow-up care can help ensure that treatment is effective and side effects are managed appropriately [47]. Inhaled steroids offer several advantages in the treatment of airway granulomas. Their localized action allows for targeted treatment directly to the airways, reducing systemic exposure and minimizing potential side effects compared to oral or systemic steroid use [15]. Inhaled steroids effectively reduce inflammation in the airways, helping to decrease the size of granulomas and alleviate associated symptoms [12]. They generally have a favorable safety profile when used as prescribed, with a lower risk of systemic side effects compared to systemic corticosteroids [48].

However, there are some disadvantages to consider. Local side effects such as throat irritation, hoarseness, and oral candidiasis can occur with inhaled steroid use, although these can be minimized with proper inhalation technique and preventative measures [49]. Prolonged or high-dose use of inhaled steroids can potentially lead to adrenal suppression, necessitating a gradual tapering-off period to avoid withdrawal symptoms [50].

Our systematic review suggests that inhaled steroids have low rates of side effects and recurrence when used for the treatment of laryngeal granulomas and vocal process granulomas. This is consistent with previous studies, such as Martins et al. (2019) and Perkins et al. (2018), which found low rates of side effects and recurrence with inhaled steroid therapy for these conditions. However, more research is needed to determine the side effects and recurrence rates of inhaled steroids for other types of airway granulomas [23,24].

Our systematic review suggests that inhaled steroids can be a safe and effective treatment option for some types of airway granulomas, such as laryngeal granulomas caused by reflux and vocal process granulomas. However, more research is needed to establish the efficacy and safety of inhaled steroids for other types of airway granulomas, such as post-intubation laryngeal granulomas and Wegener’s granulomatosis. Further high-quality studies are needed to determine the optimal dosing and duration of inhaled steroid therapy for these conditions, as well as to identify any potential side effects and recurrence rates.

## 5. Conclusions

The present systematic review sought to assess the efficacy of inhaled steroids in the treatment of airway laryngeal granulomas. The findings indicate that inhaled steroids exhibit promise as an effective therapeutic approach for managing various types of airway granulomas, encompassing laryngeal granulomas, vocal process granulomas, and intubation granulomas. Within the context of this review, it is noteworthy that a combination of inhaled steroids and complementary treatments such as proton pump inhibitors or endoscopic surgery has demonstrated efficacy in managing granulomas, often accompanied by a low incidence of adverse effects and recurrence. Inhaled steroids represent a promising and safe treatment modality for airway granulomas. However, further research is imperative to refine our comprehension of their efficacy and potential limitations. Clinical decisions regarding treatment options should be tailored to each patient, accounting for their unique medical history, presenting symptoms, and other relevant factors. This personalized approach remains pivotal in optimizing the management of airway granulomas in clinical practice.

## Figures and Tables

**Table 1:** The extraction table of the included studies.

Author and Year	Study Design	Title	Participants	Interventions	Expected Results
[23] (Martins et al., 2019)	Retrospective	Treatment of Laryngeal Granulomas	42	Inhaled steroids vs. surgical removal	In laryngeal granulomas caused by reflux, treatment with inhaled steroids and proton pump inhibitors proved to be effective, although prolonged. In postintubation and idiopathic granulomas, surgery was the best treatment.
[19] (Rimoili et al., 2018)	Systematic review	Treatment of post-intubation laryngeal granulomas: systematic review	85	Inhaled steroids	There is no evidence of high quality that proves the efficacy of any treatment for laryngeal granulomas resulting from endotracheal intubation
[28] (Nouraei et al., 2008)	Case control	Results of endoscopic surgery and intralesional steroid therapy for airway compromise due to tracheobronchial Wegener’s granulomatosis	53	Inhaled steroids	Steroid therapy and conservative endoluminal surgery is an effective strategy for treating airway compromise due to active tracheal and bronchial WG, obviating the need for airway bypass or stenting.
[24] (Perkins et al., 2018	Retrospective	Inhaled Triamcinolone With Proton Pump Inhibitor for Treatment of Vocal Process Granulomas	67	Inhaled steroids	The anti-inflammatory action of inhaled triamcinolone combined with anti-reflux proton pump inhibitors successfully treats most vocal process granulomas with low rates of side effects and recurrence.
[18] (Elshabboury, 2015)	RCT	Steroid Inhalation Versus Surgery in Treatment of Post-Intubation Granuloma	30	Inhaled steroids vs. surgery	Advise to manage intubation granuloma of the larynx mainly by inhaled steroid as a first line of treatment.
[29] (Hollis et al., 2022	Retrospective	Postoperative Inhaled Steroids Following Glottic Airway Surgery Reduces Granulation Tissue Formation	150	Inhaled steroids	Immediate postoperative use of inhaled corticosteroids seems to be a safe and effective method to prevent granulation tissue formation and subsequent surgery in patients following transoral laser airway surgery for glottic stenosis.
[26] (Teo et al., 2008)	Observational	Recurrent foreign body granuloma with airway obstruction: Is there a role for steroids	1	Steroids	Steroids are considered as an adjunct in intractable cases of foreign body granuloma.
[25] (Yoki et al., 2014)	Retrospective	Topical inhalant steroid (budesonide, Pulmicort nasal) therapy in intubation granuloma	39	Inhaled steroids	Inhaled budesonide is effective for treatment of tracheal granulation tissue in patients with tracheostomies after repair of CTS.
[27] (Karkos et al., 2014)	RCT	Vocal Process Granulomas: A Systematic Review of Treatment	75	Several treatment options	There are 6 different treatment options (single or combined) for VPG. Anti-reflux medication is the mainstay treatment and when combined with lifestyle changes and voice therapy results in the lowest recurrence rate. “Bloodless” in-office or in-theater laser techniques appear to have lower recurrence rates when compared to traditional cold steel micro laryngoscopy techniques, especially for recurrences.

## Data Availability

Data available upon request.
